# Estimating the Potential Burden of Clinically Significant Hantavirus Cases in Argentina

**DOI:** 10.1016/j.lanepe.2026.101746

**Published:** 2026-06-01

**Authors:** Younjung Kim, Christl A. Donnelly

**Affiliations:** a Department of Statistics, University of Oxford, Oxford, United Kingdom; b Pandemic Sciences Institute, University of Oxford, Oxford, United Kingdom

Cruise ships represent semi-closed environments characterised by relatively high population density, shared communal spaces, and prolonged interpersonal contacts, providing ideal settings for outbreak clusters of human-to-human-transmissible viruses to occur.^1^ We note that the recent hantavirus outbreak on the cruise ship *MV Hondius* resulted in fatalities or severe clinical signs among early cases,^2^ providing a unique statistical signal to estimate the true burden of hantavirus cases presenting severe clinical conditions (hereafter defined as ‘clinically significant hantavirus cases’) in the area in which the index case acquired infection.

By leveraging the statistical signal from the outbreak aboard *MV Hondius* alongside tourism statistics (non-resident cruise passenger counts, cruise duration, and inland travel duration), population census figures, and the time from exposure to clinical detection, we can estimate the probability of an infected non-resident cruise passenger being detected during the cruise journey and, based on this probability, the true burden of clinically significant hantavirus cases in the assumed at-risk resident population. We also note that the main hantavirus transmission season substantially overlaps with the cruise season, making our approach highly relevant from an epidemiological perspective^3^; during the 12-month period from May 2025 through April 2026, 84.9% (90 out of 106) of the reported hantavirus cases occurred during the cruise season from September 2025 through April 2026.^3^ Our approach builds upon prior research estimating the number of pandemic H1N1 influenza infections in Mexico and the number of symptomatic COVID-19 cases in Wuhan City, China. Our methodology, data, and the underlying assumptions are detailed in Supplementary Information and Supplementary File 1.

We explored baseline and alternative scenarios regarding the at-risk population, average duration of inland travel, cruise passenger totals, and the proportion of cruise passengers travelling inland prior to embarkation (Table 1). Under the baseline scenario, for the 2025–2026 cruise season from September through April, we estimated 292 (95% CI: 17–1285) clinically significant hantavirus cases among dispersed rural residents in Argentine provinces with officially reported cases since July 2019. This central estimate was 3.2 times higher (95% CI: 0.81 times lower to 14 times higher) than 90 cases reported from September 2025 through April 2026. Central estimates from the explored alternative scenarios were all greater than the reported number, despite wide confidence intervals encompassing the reported number (Table 1).

**Table 1:** Estimated numbers of clinically significant hantavirus cases during the 2025–2026 cruise season (from September through April) under alternative scenarios on the underlying factors.

	Baseline^b^	Alternative assumptions^a^, ^b^					
		Scenario A	Scenario B	Scenario C	Scenario D	Scenario E	Scenario F
Risk population^c^	Dispersed rural residents in provinces with cases	**Total rural residents in provinces with cases**	**Total rural residents in Argentina**	Dispersed rural residents in provinces with cases	Dispersed rural residents in provinces with cases	Dispersed rural residents in provinces with cases	Dispersed rural residents in provinces with cases
Duration of cruise travel	15 days	15 days	15 days	**20 days**	15 days	15 days	15 days
Duration of inland travel^d^ (a single province only, additional provinces)	8.6 days, 13.4 days	8.6 days, 13.4 days	8.6 days, 13.4 days	8.6 days, 13.4 days	**5.7 days, 8.9 days**	8.6 days, 13.4 days	8.6 days, 13.4 days
Assumptions regarding cruise passenger totals^e^	Lower bound	Lower bound	Lower bound	Lower bound	Lower bound	**No duplication**	Lower bound
Proportion of passengers travelling inland before embarkation	50%	50%	50%	50%	50%	50%	**100%**
Probability of onboard clinical detection (mean)	49.0%	49.0%	49.0%	71.7%	35.8%	49.0%	49.0%
Expected total number of cases among residents during the 2025–2026 cruise season (from September through April) (95% CI)	292.0 (16.7–1285.4)	455.1 (26.0–2003.4)	764.3 (43.6–3364.6)	199.5 (11.4–878.3)	599.0 (34.2–2637.0)	241.8 (13.8–1064.3)	146.0 (8.3–642.7)

The reported number of cases from September 2025 through April 2026 (from May 2025 through April 2026) is 90 (106).

a Alternative assumptions are highlighted in bold.

b Provinces with a least one case officially reported since July 2019.^2^

c Total rural residents are defined as individuals living in areas with fewer than 2000 inhabitants, while dispersed rural residents, a subset of total rural residents, specifically refers to those residing in the open countryside (see Supplementary Information).

d Duration of inland travel for non-resident travellers whose final destination was Ciudad Autónoma de Buenos Aires (CABA), Chubut, or Tierra del Fuego, where the cruise ports are located (see Supplementary Information).

e Lower bound: the maximum passenger count recorded at any single port for each specific ship was treated as the number of unique passengers on board; No duplication: the raw passenger totals reported across all ports were assumed to represent the number of unique passengers (see Supplementary Information).

Our analysis focused only on the main transmission season, from September through April. Therefore, if annualised for the fully comparable time period, our estimates would increase slightly. We also note that these estimates are subject to the underlying assumptions considered. Relaxing some of the key assumptions, such as lowering the probability of onboard clinical detection, reducing the proportion of cruise passengers who travelled inland prior to embarkation, and shortening the average duration of such inland travel, would result in even higher estimates. Additionally, depending on the direction and magnitude of the differences in risk between dispersed rural residents and non-resident travellers, our estimates, obtained assuming an equal daily infection risk, could have over- or under-estimated the true burden. For example, in order to argue that the observed case number reflects complete surveillance of clinically significant hantavirus cases, rural residents would need to be at a substantially lower infection risk than non-resident travellers (e.g. travellers engaging in high-risk outdoor activities). Conversely, if rural residents face a higher risk than non-resident travellers (e.g. occupational exposures among farmers), our estimates likely represent the lower bound of the true burden.

Despite these uncertainties from the underlying assumptions and wide confidence intervals, our estimates highlight the potential for substantial undetected clinically significant hantavirus infections in Argentina. Such under-detection likely stems from a non-specific prodromal phase and rapid clinical deterioration,^4^ further complicated by incomplete exposure histories. We note that our estimates correspond to the burden of hantavirus cases among rural residents with clinical presentations severe enough to trigger diagnosis, comparable to early cases on the cruise ship that collectively led to the outbreak detection. The true burden of infections is therefore likely higher, as supported by serological evidence,^5^ influenced by limited diagnostic capacity to detect mild or atypical cases and the level of risk present for urban residents. This level of potentially undetected cases underscores the need for increased efforts to enhance surveillance.

## Contributors

Younjung Kim: Conceptualisation, Investigation, Data curation, Formal analysis, Methodology, Validation, Writing–original draft, Writing–review & editing.

Christl A. Donnelly: Conceptualisation, Investigation, Funding acquisition, Formal analysis, Methodology, Validation, Supervision, Writing–review & editing.

## Data sharing statement

The study was conducted using publicly available data. All data sources are detailed and referenced in Supplementary Information and Supplementary File 1. The Excel file and R script used to estimate parameters are provided as Supplementary Files 1 and 2, respectively.

## Declaration of interests

This work is supported by grant funding (awarded to Christl A. Donnelly) from the UK National Institute for Health and Care Research (Health Protection Research Unit in Emerging and Zoonotic Infections) (grant no: HPRU200907) and the Oxford Martin School (Programme in Digital Pandemic Preparedness). Christl A. Donnelly serves for the World Health Organization R&D Blueprint in an unpaid position developing and evaluating possible vaccine and therapy effectiveness study designs. The authors declare no conflicts of interest.
