# Supplementary material for: Estimating the Potential Burden of Clinically Significant Hantavirus Cases in Argentina
Source: Lancet Reg Health Eur. 2026 Jun 1;66:101746. doi: 10.1016/j.lanepe.2026.101746 (PMC13279417; doi:10.1016/j.lanepe.2026.101746)
Supplement: Supplementary Information [file mmc3.docx]

**Supplementary Information for**

**Estimating the Potential Burden of Clinically Significant Hantavirus Cases in Argentina**

Younjung Kim^1,2^, Christl A. Donnelly^1,2^

1 Department of Statistics, University of Oxford, Oxford, United Kingdom

2 Pandemic Sciences Institute, University of Oxford, Oxford, United Kingdom

**Corresponding author**: Christl A. Donnelly

Department of Statistics, University of Oxford, 24-29 St Giles', Oxford OX1 3LB, United Kingdom, [christl.donnelly@stats.ox.ac.uk](mailto:christl.donnelly@stats.ox.ac.uk), +44 (0)1865 272860

**Data**

- The number of non-resident cruise passengers utilising the port of Ciudad Autónoma de Buenos Aires (CABA), Puerto Madryn, and Ushuaia during the 2024–2025 cruise season ^1^.
- Tourist survey from September 2024 through April 2025 on ^2^:
  - a) the final destination province
  - b) visits to provinces other than the final destination
  - c) inland travel duration stratified by whether travellers visited only the final destination or other provinces as well
- 2022 census data for provincial population size ^3^.
- 2010 census data for the proportion of rural populations ^4^.

**Underlying assumptions**

- Hantavirus infections are most commonly identified in the rural parts of South America ^5,6^. Accordingly, for the baseline scenario, we assumed that the risk of hantavirus infection was present only for rural residents dispersed in the open countryside of the Argentine provinces where cases have been officially reported since July 2019 ^7^ (see Table 1 in the main text for sensitivity analysis results from the alternative scenarios assuming all rural residents in those provinces or all rural residents in all Argentine provinces as the at-risk population).
- We assumed that rural residents and non-resident travellers experienced an equal risk of hantavirus infection per day (e.g. through leisure activities in rural areas) ^8^.
- We assumed that the outbreak on the cruise ship originated from a single index case followed by human-to-human transmission.
- We assumed that the index case of the cruise ship outbreak was exposed to the virus in Argentina.
- We assumed that a hantavirus infection will be detected in a cruise passenger if the individual’s prodromal period ends during the cruise journey.
- We assumed that the average itinerary length of non-resident travellers, captured by the international tourism survey across air, port, and land crossings ^2^, were representative of those of cruise passengers prior to embarkation.

**Data processing**

1. **Cruise passenger data**

Passenger totals published in the national maritime cruising registry (Crucerismo Marítimo) are subject to duplicate counting when cruise ships visit multiple Argentine ports (CABA, Puerto Madryn, and Ushuaia) ^1^. According to the registry’s passenger dataset by cruise ship (‘*Cruceristas por temporada, puerto y crucero*’) ^1^, 41 cruise ships visited a single Argentine port during the 2024–2025 season; passenger totals from these ships (n=426,399, 67.3%) were treated as unique passenger counts. In contrast, 12 ships visited two ports (accounting for 23,646 recorded passengers, 3.7%), and 13 ships visited all three ports (accounting for 183,386 recorded passengers, 29.0%). To minimise transit duplication for these multi-port itineraries, we extracted the maximum passenger count recorded at any single port for each specific ship. In the absence of publicly available data regarding unique passenger totals, this deduplication approach provides a lower-bound estimate of the unique passenger cohort (see Table 1 in the main text for sensitivity analysis results regarding this assumption). Based on the registry’s passenger dataset by residence status (‘*Cruceristas por temporada, mes, puerto y residencia*’) ^1^, we determined that 61.7% of passengers were non-residents, yielding an estimated total of 323,353 unique non-resident passengers across the three Argentine ports during the 2024–2025 season. We assumed this baseline figure remained constant for the 2025–2026 season. Finally, considering that CABA and Ushuaia mainly serve as the embarkation (or disembarkation) ports, rather than intermediate ports, we assumed that half of these non-resident passengers travelled inland prior to boarding their cruise ships, giving rise to 161,677 passengers used for our baseline scenario (see Table 1 in the main text for sensitivity analysis results regarding this assumption).

1. **Average duration of inland travel**

Data regarding inland travel duration were derived from the national tourism survey (Encuesta de Turismo Internacional), specifically the dataset detailing tourist overnight stays by origin and destinations visited (‘*Turistas y pernoctes según origen por provincia de destino y destinos visitados*’) ^2^. This dataset reports the duration of overnight stays for non-resident travellers in Argentina, stratified by their final destination province and whether their itineraries included additional provinces. We extracted the reported number of travel days for tourists whose final destinations were CABA, Chubut, or Tierra del Fuego, the first-order jurisdiction (capital or provinces) hosting the cruise ports. To produce a final average duration of stay, we weighted these figures by the proportion of individuals visiting exclusively the final destination province versus those visiting multiple provinces. We assumed that cruise passengers engaging in pre-embarkation inland travel would do so for this average duration, although the index case of the cruise outbreak is known to have travelled a longer duration before embarkation ^9,10^ (see Table 1 in the main text for sensitivity analysis results regarding this assumption).

1. **Average duration of cruise**

The official “2024–2025 Cruise Season Report” (Informe de Cruceros Temporada 2024–2025) for the port of Ushuaia documents that cruise durations generally fall into two categories: shorter regional trips of 3 to 7 nights, and extended voyages ranging from 10 to 30 nights, depending on the destination ^11^. Because Antarctic and international cruises account for 92% of the total passenger volume at this port ^11^, it is reasonable to assume that the vast majority of non-resident tourists engage in these longer itineraries. Furthermore, the port of CABA serves as the primary embarkation point for major international cruise routes, with Puerto Madryn typically serving as an intermediate port connecting CABA and Ushuaia ^1^. Therefore, we selected 15 days, which aligns the standard commercial itineraries departing from these ports, as the average duration of a cruse in our estimation (see Table 1 in the main text for sensitivity analysis results regarding this assumption).

**Parameter estimation**

Our work adapted approaches from prior research that estimated the number of pandemic H1N1 influenza infections in Mexico ^12^ and the number of symptomatic COVID-19 cases in Wuhan City, China ^13^. Specifically, we estimated the probability of an infected cruise passenger being detected during the cruise journey and then, based on this probability, estimated the true burden of clinically comparable hantavirus cases in the source population.

1. **Probability of onboard clinical detection**

The probability of onboard clinical detection was estimated based on the time from exposure to hospitalisation for 11 hantavirus pulmonary syndrome cases in Chile whose exposure time window was less than 48 hours ^14^. For each case, we extracted the minimum and maximum possible values directly from the original study's figure and used the calculated mean value for our analysis (Supplementary Table). Essentially, we assessed this probability based on a hypothetical scenario in which these empirical cases were infected while travelling inland and subsequently embarked on a cruise ship.

A stochastic simulation was repeated 100,000 times for each case. First, an infection day was randomly sampled from a uniform distribution between Day 0 and Day X (i.e. the average inland travel duration), assuming a constant risk of hantavirus infection during this travel period. Next, the expected clinical detection day was calculated by adding the days until hospitalisation, specific to the case, to the sampled infection day.

We defined the onboard 'cruise window' as the period immediately following inland travel: starting upon embarkation (Day X) and ending upon disembarkation (Day X + the average cruise duration). Thus, if the calculated clinical detection day fell within this specific cruise window, we assumed the case would have been detected on board. The mean proportion of onboard clinical detections was calculated across the 100,000 simulations for each case, and the mean of these probabilities was used as the final model input.

The R script used to estimate the probability of onboard clinical detection is provided in Supplementary Files.

| **Supplementary Table.** Incubation and prodrome periods of hantavirus pulmonary syndrome cases extracted from Vial et al. (2006) ^14^. | | |
| --- | --- | --- |
| Patient ID ^a^ | Incubation (mean of minimum and maximum possible) | Incubation + prodrome |
| **20** | 31.5 | 35.5 |
| **19** | 20.5 | 26.5 |
| **18** | 20.5 | 22.5 |
| **17** | 16.5 | 21.5 |
| **16** | 14.5 | 18.5 |
| **15** | 14.5 | 17.5 |
| **14** | 14.5 | 17.5 |
| **13** | 13.5 | 18.5 |
| **12** | 10.5 | 15.5 |
| **11** | 33 | 40 |
| **10** | 19 | 24 |
| 9 | 26.5 | 31.5 |
| 8 | 22.5 | 26.5 |
| 7 | 14.5 | 16.5 |
| 6 | 17 | 19 |
| 5 | 12 | 15 |
| 4 | 17 | 21 |
| 3 | 32.5 | 37.5 |
| 2 | 20.5 | 26.5 |
| 1 | 16 | 21 |
| Median | 17 | 21.5 |
| Min | 10.5 | 15.5 |
| Max | 33 | 40 |
| a Patients 10 – 20 had exposure windows less than 48 hours, and their values were used for our analysis. | | |

1. **Estimation of clinically comparable hantavirus cases among residents**

We used the probability of onboard clinical detection to estimate the expected number of clinically comparable hantavirus cases among non-resident cruise passengers, $N_{cruise}^{case}$:

$$N_{cruise}^{case}=\frac{No. of observed cases detected onboard}{Probability of onboard clinical detection}$$

Then, the expected number of clinically comparable hantavirus cases among residents in Argentina, $N_{residents}^{case}$, was obtained by extrapolating $N_{cruise}^{case}$ based on the relative total person-time of residents and non-resident cruise passengers:

$$N_{residents}^{case}=\frac{\text{Total person-time of residents during the cruise season}}{\text{Total person-time of non-resident cruise passengers}}\times N_{cruise}^{case}$$

Under the baseline assumption, the extrapolation was conducted by restricting the at-risk resident population strictly to rural residents (individuals residing in areas with fewer than 2,000 inhabitants) across Argentina. Under alternative assumptions, the extrapolation was restricted to the provinces where hantavirus cases have been officially reported since July 2019 ^7^, either among rural residents or, among them, only to dispersed rural residents (individuals residing in the open countryside), by applying their proportions from 2010 census data ^4^ to the population from 2022 census data ^3^.

Finally, confidence intervals were obtained using a profile likelihood approach based on a Poisson distribution for the single observed index case.

The Excel file used to obtain the final estimate is provided in Supplementary Files.

**Considerations for Interpreting Results**

Several additional points should be considered when interpreting our results.

- We assumed that the index case of the cruise ship outbreak was exposed to the virus in Argentina. The index case is known to have visited Neuquén ^10^, one of the Argentine provinces where hantavirus cases have been reported, and the index case’s travel history suggests potential exposure to rodents during bird-watching activities in Tierra del Fuego several days before embarkation ^9^. However, the index case also travelled to Chile and Uruguay before arriving in Argentina ^9^. This travel history indicates that given the relatively long maximum incubation period of hantavirus infection up to several weeks ^14^, the possibility that the index case was exposed elsewhere cannot be completely excluded, highlighting the need for detailed phylogenetic analysis results to better identify the most likely location of infection.
- We assumed that rural residents and non-resident travellers experienced an equal risk of hantavirus infection per day (e.g. through leisure activities in rural areas) ^8^. However, depending on the direction and magnitude of the differences in risk between dispersed rural residents and non-resident travellers, our estimates, obtained assuming an equal daily infection risk, could have over- or under-estimated the true burden. For example, in order to argue that the observed case number reflects complete surveillance of clinically significant hantavirus cases, rural residents would need to be at a substantially lower infection risk than non-resident travellers (e.g. travellers engaging in high-risk outdoor activities). Conversely, if rural residents face a higher risk than non-resident travellers (e.g. occupational exposures among farmers), our estimates likely represent the lower bound of the true burden.
- We assumed that a hantavirus infection will be detected in a cruise passenger if the individual’s prodromal period ends during the cruise journey. However, it is likely that some infections go undetected even when clinical signs are present onboard, especially if the index case does not lead to human-to-human transmission; a lower probability of onboard clinical detection would lead to a higher estimated burden of clinically significant hantavirus cases in Argentina.
- We assumed that the average itinerary length of non-resident travellers, captured by the international tourism survey across air, port, and land crossings ^2^, were representative of those of cruise passengers prior to embarkation; if cruise passengers’ duration of stay is shorter than that of general non-resident travellers, the true burden among rural residents would be estimated higher than the current estimates (Table 1 in the main text).
- Our estimates depend upon the proportion of cruise passengers who travelled inland prior to embarkation; a lower proportion of non-resident cruise passengers being potentially exposed to infection in Argentina would lead to a higher estimated burden of clinically significant hantavirus cases.

**References**

1. Dirección Nacional de Mercados y Estadística. Dataset de Crucerismo Marítimo. Sistema de Información Turística de la Argentina (SINTA), Ministerio de Turismo y Deportes; 2026.

2. Dirección Nacional de Mercados y Estadística. Encuesta de Turismo Internacional (ETI).

3. Gobierno de Argentina. Nuestro país. 2026. <https://www.argentina.gob.ar/pais>.

4. Instituto Nacional de Estadística y Censos. Población urbana y rural por provincia. Año 2010. Ministerio de Economía, República Argentina; 2010.

5. Ferro I, Bellomo CM, López W, et al. Hantavirus pulmonary syndrome outbreaks associated with climate variability in Northwestern Argentina, 1997–2017. *PLOS Neglected Tropical Diseases* 2020; **14**(11): e0008786.

6. Alonso Daniel O, Iglesias A, Coelho R, et al. Epidemiological description, case-fatality rate, and trends of Hantavirus Pulmonary Syndrome: 9 years of surveillance in Argentina. *Journal of Medical Virology* 2019; **91**(7): 1173-81.

7. Ministerio de Salud de la Nación RA. Boletín Epidemiológico Nacional N° 806 (SE 16): Ministerio de Salud de la Nación, 2026.

8. Murgue B, Domart Y, Coudrier D, et al. First reported case of imported hantavirus pulmonary syndrome in europe. *Emerg Infect Dis* 2002; **8**(1): 106-7.

9. World Health Organization. Disease Outbreak News: Hantavirus cluster linked to cruise ship travel, Multi-country. May 8 2026. <https://www.who.int/emergencies/disease-outbreak-news/item/2026-DON600>.

10. Zibell M. Tourist hotspot at 'end of the world' denies causing hantavirus outbreak. May 11 2026. https://www.bbc.co.uk/news/articles/cx21ej471g2o.

Sistema de Información Turística de la Argentina (SINTA), Ministerio de Turismo y Deportes; 2026.

11. Dirección Provincial de Puertos, Provincia de Tierra del Fuego Universidad Nacional de Tierra del Fuego. Informe Temporada de Cruceros 2024-2025: Gobierno de Tierra del Fuego, Antártida e Islas del Atlántico Sur, 2025.

12. Fraser C, Donnelly CA, Cauchemez S, et al. Pandemic Potential of a Strain of Influenza A (H1N1): Early Findings. *Science* 2009; **324**(5934): 1557-61.

13. Imai N, Dorigatti I, Cori A, Riley S, Ferguson NM. Report 1: Estimating the potential total number of novel Coronavirus cases in Wuhan City, China: MRC Centre for Global Infectious Disease Analysis, Imperial College London, 2020.

14. Vial PA, Valdivieso F, Mertz G, et al. Incubation period of hantavirus cardiopulmonary syndrome. *Emerg Infect Dis* 2006; **12**(8): 1271-3.
